# Association of SARC-F Questionnaire and Mortality in Prevalent Hemodialysis Patients

**DOI:** 10.3390/diagnostics10110890

**Published:** 2020-10-31

**Authors:** Yu-Li Lin, Jia-Sian Hou, Yu-Hsien Lai, Chih-Hsien Wang, Chiu-Huang Kuo, Hung-Hsiang Liou, Bang-Gee Hsu

**Affiliations:** 1 Division of Nephrology, Hualien Tzu Chi Hospital, Buddhist Tzu Chi Medical Foundation, Hualien 97004, Taiwan; nomo8931126@gmail.com (Y.-L.L.); simianlkive@gmail.com (J.-S.H.); hsienhsien@gmail.com (Y.-H.L.); wangch33@gmail.com (C.-H.W.); hermit.kuo@gmail.com (C.-H.K.); 2 School of Medicine, Tzu Chi University, Hualien 97004, Taiwan; 3 School of Post-Baccalaureate Chinese Medicine, Tzu Chi University, Hualien 97004, Taiwan; 4 Division of Nephrology, Department of Internal Medicine, Hsin-Jen Hospital, New Taipei City 24243, Taiwan

**Keywords:** SARC-F, sarcopenia, mortality, hemodialysis

## Abstract

Sarcopenia is common in patients undergoing chronic hemodialysis, which leads to poor outcomes. SARC-F (sluggishness, assistance in walking, rising from a chair, climb stairs, falls), a self-report questionnaire, is recommended as an easily applied tool for screening sarcopenia in older people. However, there are limited data regarding its use in patients undergoing chronic hemodialysis. Therefore, we aimed to evaluate the association between SARC-F and mortality in these patients. SARC-F questionnaire was applied in 271 hemodialysis patients (mean age 64.4 ± 14.3 years) at baseline. The association between SARC-F and mortality during a 24-month follow-up was analyzed. During this follow-up period, 40 patients (14.8%) died. The discriminative power of SARC-F score for predicting mortality was 0.716 (95% confidence interval (CI) = 0.659–0.769; *p* < 0.001). The best cut-off was a score ≥1, which provided 85.0% sensitivity, 47.2% specificity, 21.8% positive predictive value, and 94.8% negative predictive value. Kaplan–Meier curves showed that patients with SARC-F ≥ 1 exhibited a higher risk of mortality than those with SARC-F < 1 (*p* < 0.001). Moreover, a stepwise decline in survival with higher SARC-F scores was also observed. After full adjustments, SARC-F ≥ 1 was independently associated with increased mortality (hazard ratio = 2.87, 95% CI = 1.11–7.38; *p* = 0.029). In conclusion, SARC-F applied for sarcopenia screening predicted mortality in patients undergoing chronic hemodialysis.

## 1. Introduction

Sarcopenia is characterized by the progressive loss of skeletal muscle mass, strength, and physical performance during the aging process and leads to poor clinical outcomes in the geriatric population [1,2,3]. The prevalence of sarcopenia is considerably higher in patients with end-stage renal disease (ESRD) compared to the general population [4,5,6]. The reasons for chronic hemodialysis (HD) patients to develop sarcopenia are attributed to premature aging, high comorbidity burdens, nutrient loss during dialysis, inadequate protein intake, inactivity, chronic inflammation, metabolic acidosis, hormone changes, accumulation of non-dialysable uremic toxins, and myostatin and angiotensin II overexpression [7,8,9].

Dual-energy X-ray absorptiometry, computed tomography, bioelectrical impedance analysis (BIA), and dynamometer readings are among the recommended techniques for measurement of skeletal muscle mass and strength in the clinical setting [10]. However, some of these are not widely applicable because of limited feasibility in clinical practice. To our knowledge, there is no consensus as to the optimal screening tool for the early detection of sarcopenia in patients undergoing hemodialysis (HD). Therefore, an easily applied and convenient screening tool in clinical practice is urgently required. Although several validated questionnaires, such as Kidney Disease Quality of Life (KDQOL) instrument, Karnofsky Performance Status Scale, and SF-36 Health Survey, are useful to determine health-related quality of life and functional status in hemodialysis patients, these questionnaires are not specifically designed for assessing sarcopenia [11].

The validity of the SARC-F (sluggishness, assistance in walking, rising from a chair, climb stairs, falls) self-report questionnaire for the screening of sarcopenia in the geriatric population has been extensively examined in recent years [12]. SARC-F has been shown not only to correlate with skeletal muscle mass and its function but also to predict mortality in these aged adults [13,14,15,16,17]. In addition to calf circumference, the revised version of the European Working Group on Sarcopenia in Older People (EWGSOP) 2019 and the Asian Working Group for Sarcopenia (AWGS) 2019, both recommend using SARC-F questionnaire as an initial screening tool for sarcopenia in older people [18,19]. Notably, a recent cross-sectional study demonstrated associations between SARC-F and both muscle strength and physical performance in patients undergoing chronic HD [20], which implied that SARC-F might be useful for sarcopenia screening in these patients. However, the association between SARC-F and mortality in patients undergoing chronic HD has not been reported yet, and its clinical utility in these patients is largely unknown.

Hence, we conducted a prospective cohort study primarily aimed to investigate the association between SARC-F and 2-year mortality in patients undergoing chronic HD and to determine the best cut-off value for the prediction of mortality. The correlations between SARC-F and skeletal muscle mass, strength, and physical performance were also evaluated in a subgroup of the participants.

## 2. Materials and Methods 

### 2.1. Setting and Participants

This prospective cohort study was conducted at a medical center in Hualien, Taiwan, and was approved by the Institutional Review Board of Tzu Chi Hospital (IRB104-84-B, approved date: 1 September 2015). In July 2018, all patients aged >20 years who underwent HD for at least three months in our HD center were recruited to the study. Among these 277 patients undergoing chronic HD, 271 (97.8%) patients were enrolled. The patients were followed until death, transplantation, transfer to other dialysis units, or end of the study in July 2020.

We collected demographic data, dialysis duration, underlying causes for dialysis, and comorbid diseases via chart review. Cardiovascular diseases included histories of coronary artery disease, acute myocardial infarction, congestive heart failure, and arrhythmias; chronic liver disease included chronic hepatitis and liver cirrhosis. The Charlson comorbidity index (CCI) was adopted to evaluate these high comorbidity burdens [21]. A subgroup of 100 participants without amputated limb(s), active malignancy, unsteady gait, or bed-ridden status was invited to undergo skeletal muscle mass, muscle strength, and physical performance measurements. Written informed consent was obtained from all participants, and all methods were performed in accordance with the relevant guidelines and regulations.

### 2.2. SARC-F

SARC-F was assessed at baseline, which is a five-item self-report questionnaire that includes the following: strength, how much difficulty do you have in lifting and carrying 10 lb?; assistance with walking, how much difficulty do you have walking across a room?; rise from a chair, how much difficulty do you have transferring from a chair or bed?; climb stairs, how much difficulty do you have climbing a flight of 10 stairs?; falls, how many times have you fallen in the past year? Each component has three severity levels ranging from zero (no difficulty or no fall) to two (great difficulty or more than four falls). A total score in the range of 0–10 was assigned to each patient [12,22]. 

### 2.3. Laboratory Tests

Fasting blood samples (~5 mL) were collected before HD sessions, according to standardized procedures. Around 0.5 mL blood samples were used for blood cell counting (Sysmex SP-1000i, Sysmex American, Mundelein, IL, USA), and the remaining volumes were immediately centrifuged for biochemical analysis within 1 h of collection. Serum concentrations of blood urea nitrogen (BUN), creatinine, albumin, phosphorus, and C-reactive protein were determined using an autoanalyzer (Siemens Advia 1800, Siemens Healthcare GmbH, Erlangen, Germany). The fractional clearance index for urea (Kt/V) was calculated using a single-compartment dialysis urea kinetic model. Daily protein intake was estimated as the normalized protein nitrogen appearance rate (nPNA) using the three-point method [23]. 

### 2.4. Assessment of Body Composition, Muscle Strength, and Physical Performance

Dry weight and height data for BMI calculations were collected for all participants. A subgroup of participants was invited to take part in mid-arm muscular circumference (MAMC), skeletal muscle mass, fat tissue mass, calf circumference, handgrip strength, hip flexion, and knee extension strength, and 6-m usual gait speed measurements and also to carry out the repeated sit-to-stand test five times. 

After measuring the mid-arm circumference (MAC) and the triceps skinfold (TSF), the MAMC was calculated based on the following formula: MAMC = MAC − π × TSF. Calf circumference was measured at the point of the greatest circumference of the calf. Skeletal muscle and fat tissue masses (kg) were measured using a tetrapolar device (Biodynamics^®^ BIA 450 Bioimpedance Analyzer, Seattle, WA, USA), which delivered an electrical current of 800 μA at 50 kHz [24]. Skeletal muscle and fat tissue masses were normalized for squared height and defined as skeletal muscle index and fat tissue index, respectively. 

We assessed the maximum handgrip strength (HGS) in bilateral hands using a dynamometer (Jamar Plus Digital Hand Dynamometer, SI Instruments Pty. Ltd., Hilton, Australia). Patients were instructed to grip the dynamometer with their maximum strength in a standing position with the arm bent at a right angle at the elbow and held at the side of the body [25]. Muscle strength of lower extremities, included hip flexion and knee extension, were assessed in a sitting position with knee flexed to 90 degrees, using a portable Force Evaluation and Testing (FET) dynamometer (MicroFET2^®^, Hogan Health Industries, Inc., UT, USA) [26,27]. During the test period of 5 s, the dynamometer pad was stabilized by the operator’s hand, and patients were instructed to push against the dynamometer pad by attempting to raise legs and straighten their knees, respectively. Patients were asked to increase force gradually to maximum voluntary effort. Three measurements of upper and lower extremities muscle strength were performed, with a rest period of 1 min, and the average values of each side were recorded for analysis.

The usual gait speed was measured in patients walking for 6 m on a flat, straight path from a static start. We calculated gait speed by dividing the distance traveled by the time taken to cover that distance. A repeated sit-to-stand test was performed to evaluate lower extremity strength. Patients were instructed to stand up from a seated position five times consecutively with their arms folded across their chests. The total time required to complete the test was recorded. The test was performed twice, and the average time value was used for analysis. 

All of the above measurements were performed before the start of the HD session by the same trained operator. 

### 2.5. Statistical Analysis

Continuous variables between groups were expressed as mean ± standard deviation or median (interquartile range) and compared using the independent t-test or Mann–Whitney U test based on the normality of the variables tested using the Kolmogorov–Smirnov test. Categorical variables were expressed as absolute (n) and relative frequency (%) and compared using the χ^2^ or Fisher’s exact test. 

To evaluate the correlation of SARC-F with skeletal muscle mass, strength, and physical performance, a total of at least 85 patients should be enrolled, assuming to detect a modest correlation (correlation coefficient 0.3) between total SARC-F scores and measured variables [14], with an alpha level of 0.05 and a power of 80%. Finally, one hundred patients were assessed. Given the right-skewed distribution of the total SARC-F, Spearman’s rank correlation coefficient was adopted. Receiver operating characteristic curves were constructed to predict 24-month mortality and to assess the diagnostic value of SARC-F scores on low skeletal muscle index, handgrip strength weakness, poor physical performance, possible sarcopenia, and sarcopenia. The areas under curves (AUC), cut-offs, sensitivity, specificity, positive predictive value (PPV), and negative predictive value (NPV) were established. Low skeletal muscle index was defined as skeletal muscle index <16.5 kg/m^2^ in men and <14.2 kg/m^2^ in women, based on the 10th percentile values of 8959 Chinese adults [28]. The cut-off values for defining handgrip strength weakness, slow gait speed, and poor sit-to-stand performance were derived from the criteria of AWGS 2020 [19]. Possible sarcopenia was diagnosed as the presence of either handgrip strength weakness or poor physical performance. Sarcopenia was diagnosed based on the presence of low skeletal muscle index with either handgrip strength weakness, slow gait speed, or poor physical performance [19]. 

In the overall survival analysis, we defined censored data as patients who were lost to follow-up, who received renal transplantation, or who did not experience mortality events during the follow-up period. Kaplan–Meier analyses with log-rank tests were used to compare the overall survival rates between two SARC-F groups, divided based on the best cut-off to predict mortality. Patients were further stratified into four groups according to the different total SARC-F scores to evaluate whether different survival impacts existed among groups. Cox-proportional hazard models were used to evaluate SARC-F as a predictor for overall mortality. The variables with significant differences between survival and non-survival were adopted as covariates.

Data were analyzed using SPSS for Windows (version 19.0, IBM Corp., Armonk, NY, USA) and Stata (version 12.0, Stata Corp., College Station, TX, USA), and *p* < 0.05 was considered statistically significant.

## 3. Results

**Figure 1:**
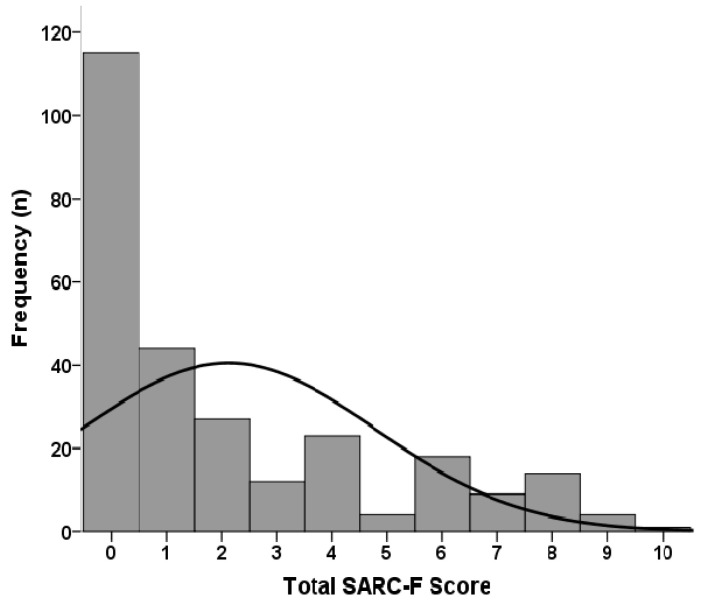
SARC-F distribution among 271 hemodialysis patients.

The mean age of these 271 patients was 64.4 ± 14.3 years, and the median dialysis duration was 4.0 (1.5–7.9) years. The main underlying causes of ESRD were diabetes mellitus (DM) (46.9%) and chronic glomerulonephritis (GN) (29.2%). The demographic and clinical characteristics of the entire cohort, categorized by gender, are presented in Table 1. Compared with females, males had shorter HD duration (*p* = 0.003), more patients had DM (*p* = 0.024), less patients had chronic GN (*p* = 0.015), lower SARC-F score (*p* = 0.016), Kt/V (*p* < 0.001), and higher serum creatinine (*p* < 0.001). The distribution of SARC-F among our patients is depicted in Figure 1, which revealed a right-skewed distribution.

The Spearman correlations of SARC-F with basic characteristics, Charlson comorbidity index, body composition, muscle strength, and physical performance are shown in Table 2. SARC-F scores were positively correlated with age (*r =* 0.405, *p* < 0.001) and Charlson comorbidity index (*r =* 0.273, *p* < 0.001). Among the parameters of muscle strength and physical performance, SARC-F scores were negatively correlated with handgrip strength (*r =* −0.284, *p* = 0.004 for the right hand; *r =* −0.302, *p* = 0.002 for the left hand), hip flexion strength (*r =* −0.236, *p* = 0.018 for the right hip; *r =* −0.301, *p* = 0.002 for the left hip), knee extension strength (*r =* −0.341, *p* = 0.001 for the right knee; *r =* −0.303, *p* = 0.002 for the left knee), and 6 m gait speed (*r =* −0.339, *p* = 0.001). It was positively and marginally correlated with repeated sit-to-stand test (*r =* 0.199, *p* = 0.058). However, among the parameters of body composition, SARC-F scores were only negatively correlated with MAMC (*r =* −0.253, *p* = 0.011) and calf circumference (*r =* −0.235, *p* = 0.019), but not with skeletal muscle index (*r =* −0.128, *p* = 0.203) and fat tissue index (*r =* 0.133, *p* = 0.187).

The diagnostic values of SARC-F scores on low skeletal muscle index, handgrip strength weakness, poor physical performance, possible sarcopenia, and sarcopenia among 100 participants are established in Table 3. SARC-F scores had fair discriminative powers for the prediction of low skeletal muscle index (area under the curve (AUC) = 0.658, 95% confidence interval (CI) = 0.556–0.750), handgrip strength weakness (AUC = 0.651, 95% CI = 0.549–0.744), slow gait speed (AUC = 0.685, 95% CI = 0.584–0.774), poor sit-to-stand performance (AUC = 0.656, 95% CI = 0.554–0.748). In together, the discriminative powers for possible sarcopenia and sarcopenia were 0.671 (95% CI = 0.570–0.762) and 0.694 (95% CI = 0.593–0.782). The best cut-off for predicting sarcopenia was a score ≥1, which provided 71.4% sensitivity and 65.6% specificity. The sensitivity, specificity, PPV, and NPV using a cut-off value of ≥4, which was recommended originally in the geriatric population, are shown in Supplementary Table S1. Notably, using this original cut-off value yielded considerably lower sensitivity.

**Figure 2:**
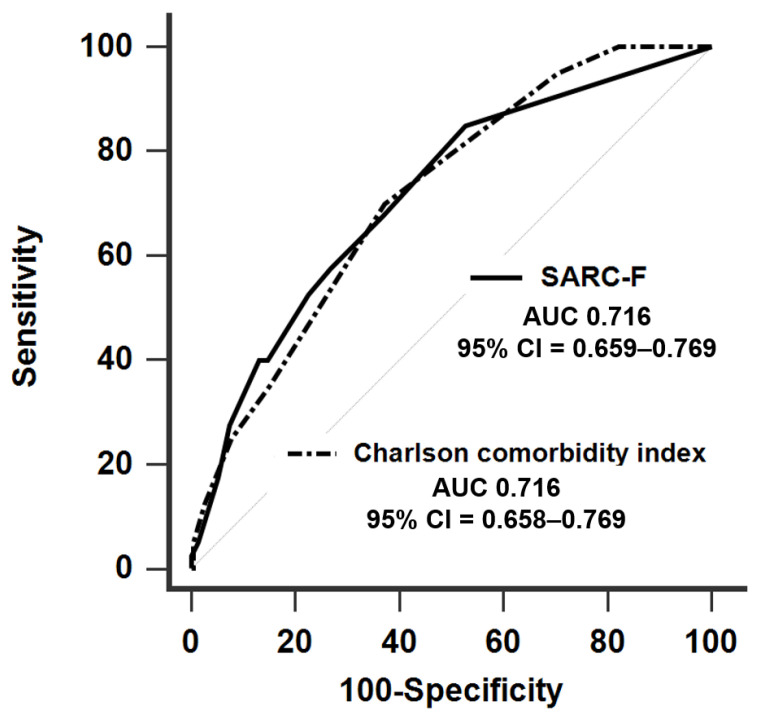
Receiver operating characteristic curves of SARC-F and Charlson comorbidity index on the prediction of mortality in 271 prevalent hemodialysis patients.

During the 24-month follow-up period, 12 patients (4.4%) were lost to follow-up, 40 patients (14.8%) died. A comparison of ROC curve between SARC-F and Charlson comorbidity index for the prediction of mortality is depicted in Figure 2, which showed similar discriminative powers (AUC 0.716, 95% CI = 0.659–0.769, *p* < 0.001 for SARC-F; AUC 0.716, 95% CI = 0.658–0.769, *p* < 0.001 for Charlson comorbidity index).

The best cut-off of SARC-F score to predict mortality was ≥1, which provided 85.0% sensitivity and 47.2% specificity. The PPV and NPV were 21.8% and 94.8%, respectively. The predictive validity of SARC-F score on mortality using different cut-off values is also shown in Supplementary Table S2. 

**Figure 3:**
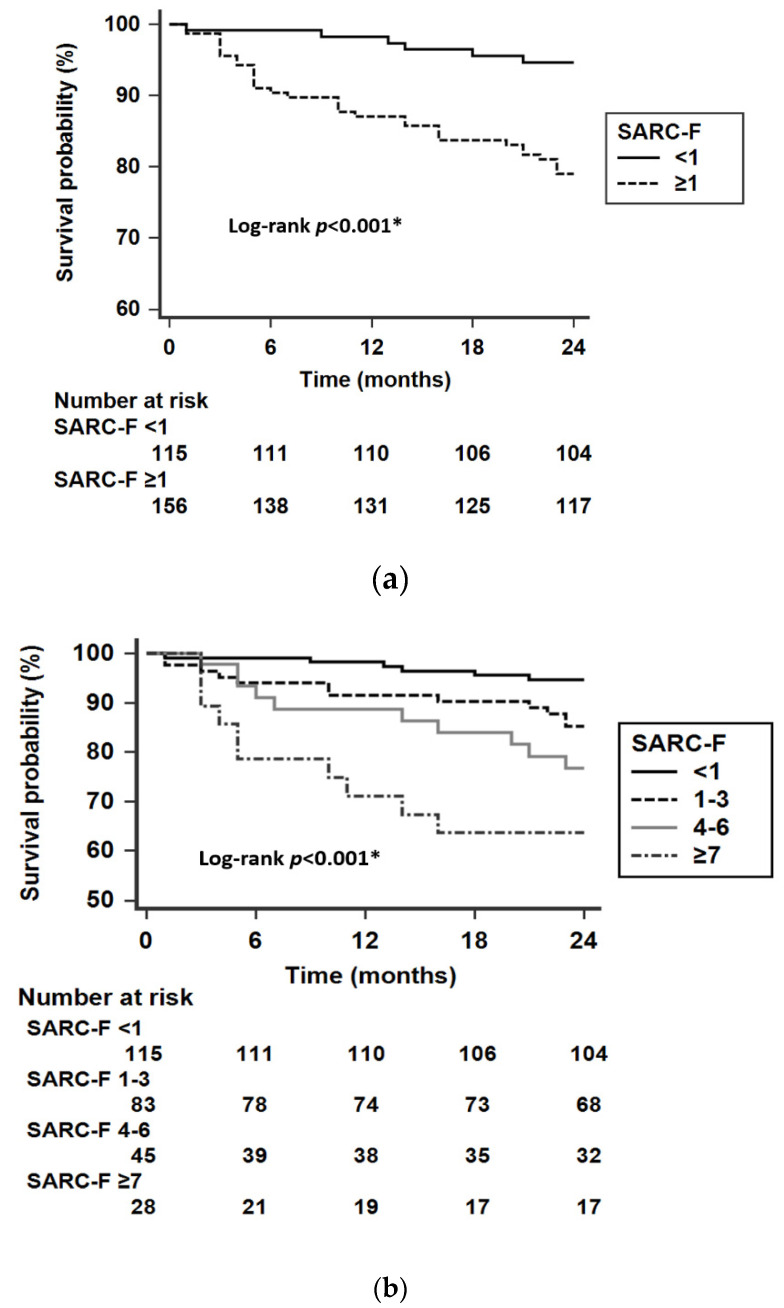
Kaplan-Meier survival analysis of study population: (**a**) patients with and without SARC-F ≥1 (**b**) patient groups stratified by different levels of total SARC-F score. * *p* < 0.05 was considered statistically significant.

Figure 3 shows the Kaplan–Meier survival curve of patients undergoing chronic HD, stratified by SARC-F score. Patients with SARC-F ≥ 1 exhibited a higher risk of mortality than those with SARC-F < 1 (*p* < 0.001). Furthermore, Kaplan–Meier curves showed a stepwise decline in survival with higher SARC-F scores (*p* < 0.001). 

Table 4 shows the comparisons between survival and non-survival patients. Age, the prevalence of cardiovascular disease, Charlson comorbidity index, SARC-F, Kt/V, and C-reactive protein were higher among non-survival patients, while BMI, serum albumin, creatinine, phosphorus, and nPNA were higher among survival patients. 

Table 5 shows the Cox-proportional hazards models for SARC-F and mortality. In crude models, higher SARC-F was associated with increased mortality, either treated as continuous (hazard ratio (HR) = 1.26, 95% CI, 1.15–1.39; *p* < 0.001) or categorical variables (HR = 4.58, 95% CI, 1.92–10.91; *p* = 0.001). After full adjustments, these associations remained significant when SARC-F was treated as a categorical variable (HR = 2.87, 95% CI, 1.11–7.38; *p* = 0.029). 

## 4. Discussion

To the best of our knowledge, this is the first study to evaluate the association between SARC-F and mortality in patients undergoing chronic HD. We found that patients who had SARC-F ≥ 1 were associated with an increase of 2-year mortality in both crude and adjusted Cox models. In addition, stratified by four groups according to different levels of SARC-F scores, patients in higher SARC-F groups yielded a progressive increment in the risk of mortality, which indicated that SARC-F could serve as a useful tool for risk stratification. 

The association between SARC-F and mortality has been confirmed in a large-scale longitudinal study, which is comprised of African American Health (AAH), Baltimore Longitudinal Study of Aging (BLSA), and National Health and Nutrition Examination Survey (NHANES) cohorts [15]. In community-dwelling senior Taiwanese, SARC-F was associated with subsequent quality of life, overall hospitalization, and 4-year mortality [14]. Consistent with these studies, we showed a close association between SARC-F and mortality in patients undergoing chronic HD. 

While SARC-F ≥ 4 has been consistently recommended as a cut-off for the screening of sarcopenia in the geriatric population, this cut-off value may be different in chronic HD patients. When we applied this cut-off value in our HD patients, it yielded low sensitivity, either in the prediction of mortality or sarcopenia, which was similar to previous reports in the geriatric population [13,29,30,31]. This may hinder its use in clinical practice to early detection of patients with mild to moderate sarcopenia. Instead, SARC-F ≥ 1 appeared to be the best cut-off value for the prediction of sarcopenia and mortality, which increased the sensitivity. 

Regarding the diagnostic validation of SARC-F, Yamamoto et al. reported an association between SARC-F and physical function in HD patients [20]. The AUCs of the SARC-F questionnaire for low handgrip strength, weak leg strength, slow gait speed, and low short physical performance battery score were 0.77, 0.76, 0.84, and 0.87. These discriminative powers indicated a good diagnostic performance of SARC-F for identifying HD patients with a physical disability. Similarly, the associations of SARC-F with physical performance, including handgrip strength, lower extremities strength, and gait speed, were confirmed in our study. In addition, we extended its application on the prediction of sarcopenia, defined as the presence of low skeletal muscle mass with either handgrip strength weakness or poor physical performance. The AUCs for sarcopenia was 0.694. A modified version of SARC-F may be needed to enhance its diagnostic performance in chronic HD patients. Nevertheless, the high NPV using a cut-off value of ≥1, which is 96.8%, suggested that the SARC-F questionnaire may be considered as an initial test for screening sarcopenia in chronic HD patients. 

To the best of our knowledge, this is the first study to report the association between SARC-F and mortality in patients undergoing chronic HD. In this study, a total of 97.8% of patients in our HD center were assessed using SARC-F, and only 4.4% of patients were lost during the follow-up period, which ensured the validity of the study. However, several limitations must be acknowledged. First, the follow-up duration was relatively short, and the low event rates in mortality could affect the precision of estimation. Second, the measurement of skeletal muscle mass may be overestimated by the hydration status of patients undergoing HD. Third, regarding the diagnostic performance of SARC-F on sarcopenia and its individual criteria, only a limited number of patients were enrolled. Thus, the results may be regarded as preliminary. Finally, this study was conducted in a single medical center, and our results may not be generalized to the overall HD population. 

In conclusion, SARC-F appears to be a clinic-friendly and easily-applied tool to predict mortality in patients undergoing chronic HD. However, whether SARC-F is a relevant screening tool for uremic sarcopenia in chronic HD patients should be determined in further large-scale studies. 

## Figures and Tables

**Table 1:** Clinical characteristics of the 271 hemodialysis patients and comparison between males and females.

Characteristics	All Patients (*n* = 271)	Men (*n* = 151)	Women (*n* = 120)	*p*
**Demographics**				
Age (years)	64.4 ± 14.3	63.0 ± 13.8	66.1 ± 14.8	0.081
HD duration (years)	4.0 (1.5–7.9)	3.4 (1.4–6.0)	4.7 (1.8–11.4)	0.003 *
**Causes of ESRD, *n* (%)**				
DM	127 (46.9)	80 (53.0)	47 (39.2)	0.024 *
Chronic GN	79 (29.2)	35 (23.2)	44 (36.7)	0.015 *
Others	65 (24.0)	36 (23.8)	29 (24.2)	0.950
**Comorbid conditions, *n* (%)**				
Cardiovascular disease	100 (36.9)	59 (39.1)	41 (34.2)	0.406
Chronic liver disease	46 (17.0)	25 (16.6)	21 (17.5)	0.837
Malignancy	31 (11.4)	16 (10.6)	15 (12.5)	0.625
Stroke	20 (7.4)	11 (7.3)	9 (7.5)	0.946
PAD	21 (7.7)	14 (9.3)	7 (5.8)	0.293
Dementia	12 (4.4)	5 (3.3)	7 (5.8)	0.316
**Charlson comorbidity index**	4 (3–5)	4 (4–5)	4 (3–5)	0.245
**SARC-F score**	1 (0–4)	1 (0–2)	2 (0–4)	0.016 *
**Examination**				
BMI (Kg/m^2^)	24.7 ± 4.8	25.1 ± 4.3	24.1 ± 5.4	0.126
Kt/V (Gotch)	1.31 (1.18–1.46)	1.21 (1.13–1.34)	1.41 (1.31–1.59)	<0.001 *
Hemoglobin (g/dL)	10.3 (9.6–10.9)	10.3 (9.5–11.1)	10.3 (9.6–10.8)	0.148
Albumin (g/dL)	4.3 (3.9–4.6)	4.3 (4.0–4.6)	4.3 (3.9–4.5)	0.180
BUN (mg/dL)	62 (51–74)	64 (52–74)	61 (51–72)	0.291
Creatinine (mg/dL)	9.2 ± 2.3	9.9 ± 2.4	8.2 ± 1.6	<0.001 *
Phosphorus (mg/dL)	4.8 (3.8–5.7)	4.9 (3.6–5.7)	4.8 (3.9–5.7)	0.608
C-reactive protein (mg/dL)	0.36 (0.09–0.87)	0.39 (0.10–0.89)	0.29 (0.08–0.86)	0.371
nPNA (g/kg/day)	0.97 (0.82–1.11)	0.95 (0.80–1.09)	0.99 (0.85–1.16)	0.249

HD, hemodialysis; ESRD, end-stage renal disease; DM, diabetes mellitus; GN, glomerulonephritis; PAD, peripheral artery disease; BMI, body mass index; Kt/V, fractional clearance index for urea; BUN, blood urea nitrogen; nPNA, normalized protein nitrogen appearance. * *p* < 0.05 is considered statistically significant, comparing differences between males and females.

**Table 2:** Spearman correlation of total SARC-F with basic characteristics, Charlson comorbidity index, body composition, muscle strength, and physical performance.

Variables	SARC-F	
	*r*	*p*
**Basic characteristics ^a^**		
Age (years)	0.405	<0.001 *
HD duration (years)	0.006	0.919
**Charlson comorbidity index ^a^**	0.273	<0.001 *
**Body composition ^b^**		
MAMC (cm)	−0.253	0.011 *
Skeletal muscle index (Kg/m^2^)	−0.128	0.203
Fat tissue index (Kg/m^2^)	0.133	0.187
Calf circumference (cm)	−0.235	0.019 *
**Muscle strength ^b^**		
Handgrip strength (Kg)		
Right	−0.284	0.004 *
Left	−0.302	0.002 *
Hip flexion strength (Kg-force)		
Right	−0.236	0.018 *
Left	−0.301	0.002 *
Knee extension strength (Kg-force)		
Right	−0.341	0.001 *
Left	−0.303	0.002 *
**Physical performance**		
6 m gait speed (m/s) ^b^	−0.339	0.001 *
Repeated sit-to-stand test (s) ^c^	0.199	0.058

^a^*n* = 271, ^b^ *n* = 100, ^c^ *n* = 92, 8 patients did not perform the test due to orthostatic dizziness. D, hemodialysis; MAMC, mid-arm muscular circumference. * *p* < 0.05 was considered statistically significant.

**Table 3:** The best cut-offs of SARC-F score on low skeletal muscle index, handgrip strength weakness, poor physical performance, possible, and definite sarcopenia among 100 hemodialysis patients.

	AUC (95% CI)	Cut-Off	Sen (%)	Spe (%)	PPV (%)	NPV (%)
Low skeletal muscle index ^a^	0.658 (0.556–0.750)	≥1	66.7	65.9	16.2	95.2
Handgrip strength weakness ^b^	0.651 (0.549–0.744)	≥2	36.6	91.5	75.0	67.5
Slow gait speed ^c^	0.685 (0.584–0.774)	≥1	56.1	76.3	62.2	71.4
Poor sit-to-stand test ≥12 s ^d^	0.656 (0.554–0.748)	≥1	49.1	77.8	73.0	55.6
Possible sarcopenia ^e^	0.671 (0.570–0.762)	≥1	47.7	82.9	83.8	46.0
Sarcopenia ^f^	0.694 (0.593–0.782)	≥1	71.4	65.6	13.5	96.8

^a^ Skeletal muscle index below the sex-specific 10th percentile of the reference population (<16.5 Kg/m^2^ in male; <14.2 Kg/m^2^ in female); ^b^ Maximum handgrip strength of both hands <28 kg in male and <18 kg in female; ^c^ 6 m gait speed <1.0 m/s; ^d^ Repeated sit-to-stand test ≥12 s; ^e^ The presence of either handgrip strength weakness or poor sit-to-stand test; ^f^ The presence of low skeletal muscle index with either handgrip strength weakness, slow gait speed, or poor sit-to-stand test; AUC, area under the curve; CI, confidence interval; Sen, sensitivity; Spe, specificity; PPV, positive predictive value; NPV, negative predictive value.

**Table 4:** Comparison between clinical characteristics of survival and non-survival patients.

Characteristics	Survival (*n* = 231)	Non-Survival (*n* = 40)	*p*
**Demographics**			
Age (years)	63.1 ± 14.1	71.7 ± 13.5	<0.001 *
Male (%)	129 (55.8)	22 (55.0)	0.921
HD duration (years)	4.1 (1.5–8.2)	4.0 (1.8–7.2)	0.715
**Causes of ESRD, *n* (%)**			
DM	105 (45.5)	22 (55.0)	0.264
Chronic GN	69 (29.9)	10 (25.0)	0.531
Others	57 (24.7)	8 (20.0)	0.523
**Comorbid conditions, *n* (%)**			
Cardiovascular disease	78 (33.8)	22 (55.0)	0.010 *
Chronic liver disease	39 (16.9)	7 (17.5)	0.924
Malignancy	23 (10.0)	8 (20.0)	0.065
Stroke	18 (7.8)	2 (5.0)	0.533
PAD	15 (6.5)	6 (15.0)	0.063
Dementia	8 (3.5)	4 (10.0)	0.064
**Charlson comorbidity index**	4 (3–5)	5 (4–7)	<0.001 *
**SARC-F**			
Total score	1 (0–3)	4 (1–7)	<0.001 *
SARC-F ≥ 1, *n* (%)	122 (52.8)	34 (85.0)	<0.001 *
**Examination**			
BMI (Kg/m^2^)	25.0 ± 4.8	22.6 ± 4.4	0.004 *
Kt/V (Gotch)	1.30 (1.18–1.40)	1.42 (1.23–1.61)	0.013 *
Hemoglobin (g/dL)	10.3 (9.7–10.9)	9.8 (9.0–10.6)	0.076
Albumin (g/dL)	4.3 (4.0–4.6)	3.9 (3.7–4.2)	<0.001 *
BUN (mg/dL)	62 (52–74)	62 (47–72)	0.403
Creatinine (mg/dL)	9.4 ± 2.2	7.7 ± 1.9	<0.001 *
Phosphorus (mg/dL)	4.9 (3.9–5.8)	4.4 (3.2–5.0)	0.022 *
C-reactive protein (mg/dL)	0.32 (0.08–0.83)	0.68 (0.29–1.22)	0.003 *
nPNA (g/kg/day)	0.99 (0.83–1.13)	0.90 (0.72–1.05)	0.039 *

HD, hemodialysis; ESRD, end-stage renal disease; DM, diabetes mellitus; GN, glomerulonephritis; PAD, peripheral artery disease; BMI, body mass index; Kt/V, fractional clearance index for urea; BUN, blood urea nitrogen; nPNA, normalized protein nitrogen appearance. * *p* < 0.05 is considered statistically significant, comparing differences between survival and non-survival patients.

**Table 5:** Cox-proportional hazards model for mortality in 271 hemodialysis patients according to the SARC-F score as a continuous or categorical variable.

Model	SARC-F (per 1 Unit of Increase)		SARC-F ≥ 1	
	HR (95% CI)	*p*	HR (95% CI)	*p*
Crude model	1.26 (1.15–1.39)	<0.001 *	4.58 (1.92–10.91)	0.001 *
Model 1	1.22 (1.10–1.36)	<0.001 *	3.55 (1.44–8.74)	0.006 *
Model 2	1.15 (1.02–1.29)	0.020 *	3.00 (1.21–7.43)	0.018 *
Model 3	1.12 (0.98–1.29)	0.110	2.87 (1.11–7.38)	0.029 *

Model 1: adjusted for age and sex. Model 2: Adjusted for Model 1 plus cardiovascular disease, Charlson comorbidity index, and body mass index. Model 3: Adjusted for Model 2 plus Kt/V, albumin, creatinine, phosphorus, C-reactive protein, and nPNA. * *p* < 0.05 was considered statistically significant.

## Supplementary Materials

The following are available online at https://www.mdpi.com/2075-4418/10/11/890/s1, Table S1: The diagnostic performances of SARC-F, using cut-off value of ≥4; Table S2: Predictive validity of SARC-F score on 24-month mortality, using different cut-off values.
