# Supplementary material for: Association of SARC-F Questionnaire and Mortality in Prevalent Hemodialysis Patients
Source: Diagnostics (Basel). 2020 Oct 31;10(11):890. doi: 10.3390/diagnostics10110890 (PMC7693515; doi:10.3390/diagnostics10110890)
Supplement: Supplementary file 1 [file diagnostics-10-00890-s001.pdf]

SUPPLEMENTARY TABLE 1. The diagnostic performances of SARC-F, using cut-off value of ≥ 4.

	Sen (%)	Spe (%)	PPV (%)	NPV (%)
Low skeletal muscle index [a]	11.1	91.2	11.1	91.2
Handgrip strength weakness [b]	17.1	96.6	77.8	62.6
Slow gait speed [c]	19.5	98.3	88.9	63.7
Poor sit-to-stand test ≥ 12s [d]	16.4	100.0	100.0	49.5
Possible sarcopenia [e]	13.9	100.0	100.0	38.5
Sarcopenia [f]	14.3	91.4	11.1	93.4

[a] Skeletal muscle index below the sex-specific 10th percentile of reference population

[b] Maximum handgrip strength of both hands <28 kg in male and < 18 kg in female

[c] 6-meter gait speed <1.0 m/s

[d] Repeated sit-to-stand test ≥ 12s

[e] The presence of either handgrip strength weakness or sit-to-stand test ≥ 12s

[f] The presence of low skeletal muscle index with either handgrip strength weakness, slow gait speed, or poor sit-to-stand test

Sen, sensitivity; Spe, specificity; PPV, positive predictive value; NPV, negative predictive value.

SUPPLEMENTARY TABLE 2. Predictive validity of SARC-F score on 24-month mortality, using different cut-off values.

Cut-off values	Sen (%)	Spe (%)	PPV (%)	NPV (%)
≥ 1¶	85.0	47.2	21.8	94.8
≥ 2	67.5	63.2	24.1	91.8
≥ 3	57.5	73.2	27.1	90.9
≥ 4	52.5	77.5	28.8	90.4
≥ 5	40.0	85.3	32.0	89.1
≥ 6	40.0	87.0	34.8	89.3
≥ 7	27.5	92.6	39.3	88.1
≥ 8	17.5	94.8	36.8	86.9
≥ 9	5.0	98.7	40.0	85.7

¶Best cut-off

Sen, sensitivity; Spe, specificity; PPV, positive predictive value; NPV, negative predictive value.
